# Supplementary material for: Chemico-Physical Properties of Some 1,1′-Bis-alkyl-2,2′-hexane-1,6-diyl-bispyridinium Chlorides Hydrogenated and Partially Fluorinated for Gene Delivery
Source: Molecules. 2023 Apr 20;28(8):3585. doi: 10.3390/molecules28083585 (PMC10145364; doi:10.3390/molecules28083585)
Supplement: Supplementary file 1 [file molecules-28-03585-s001.zip › molecules-2266406-supplementary.pdf]

SUPPORTING INFORMATION

Chemico-Physical Properties of Some 1,1′-Bis-alkyl-2,2′-hexane-1,6-diyl-bispyridinium Chlorides Hydrogenated and Partially Fluorinated for Gene Delivery

Michele Massa [1], Mirko Rivara [2], Thelma A. Pertinhez [3], Carlotta Compari [2], Gaetano Donofrio [4], Luigi Cristofolini [5], Davide Orsi [5], Valentina Franceschi [4] and Emilia Fisicaro [2,*]

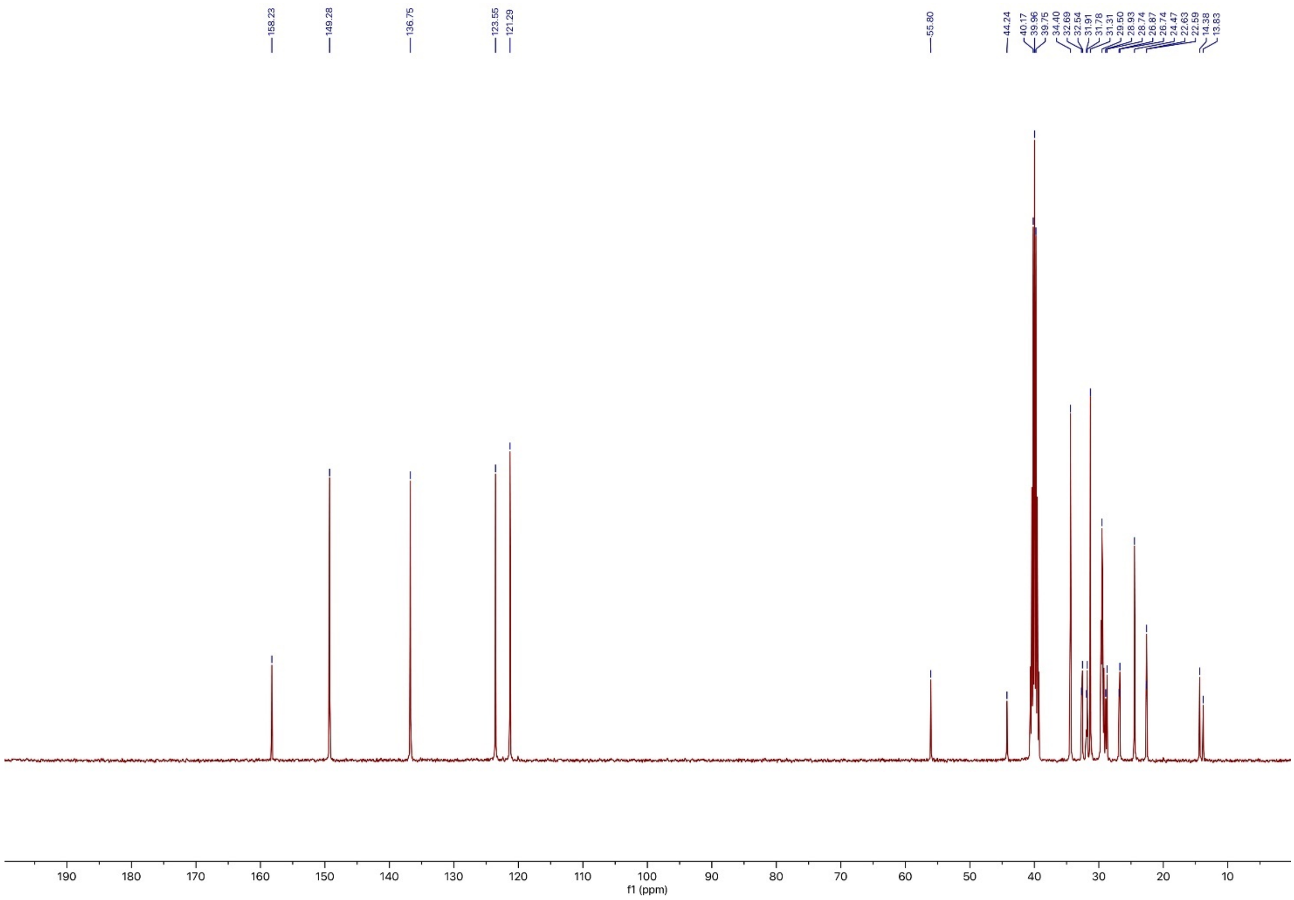

Figure S1. ^{13}C-NMR of 1,1′-bis-hexadecyl-2,2′-hexamethylenebispyridinium chloride (GP12_6).

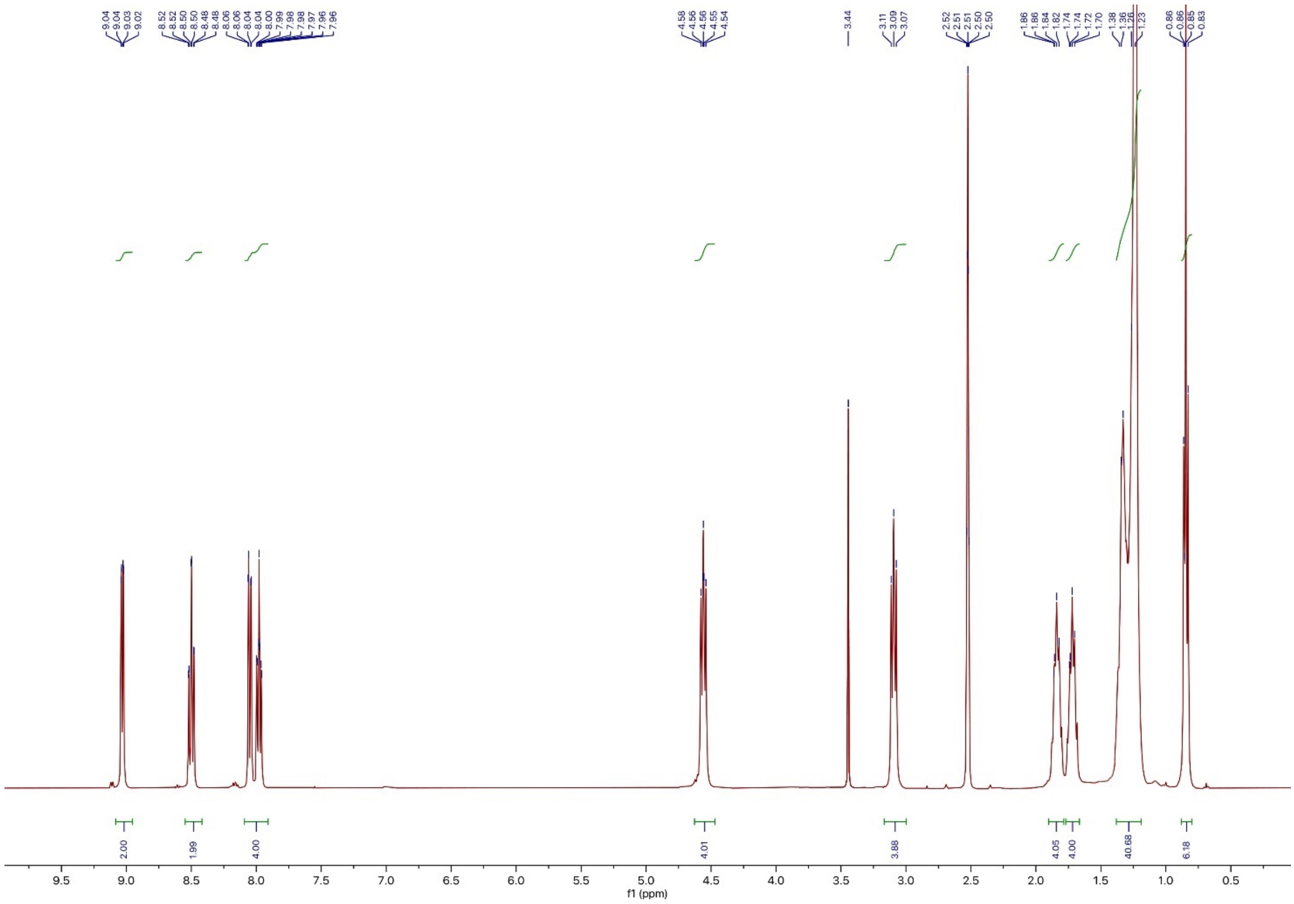

Figure S2. ^{1}H-NMR of 1,1′-bis-dodecyl-2,2′-hexamethylenebispyridinium chloride (GP12_6).

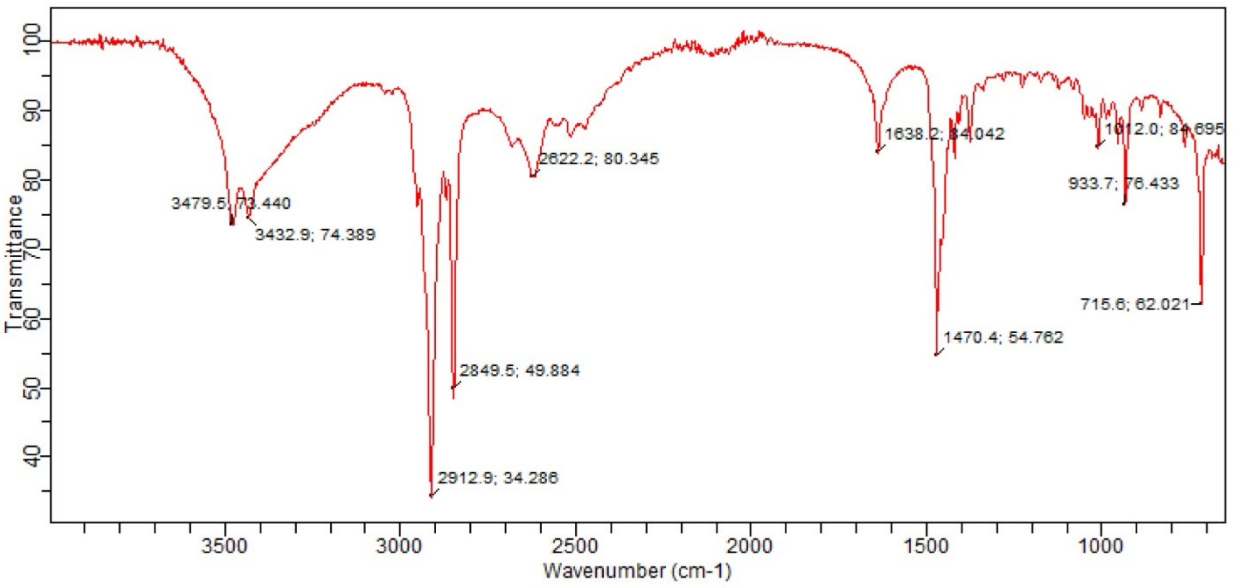

Figure S3. IR spectrum of 1,1′-bis-dodecyl-2,2′-hexamethylenebispyridinium chloride (GP12_6).

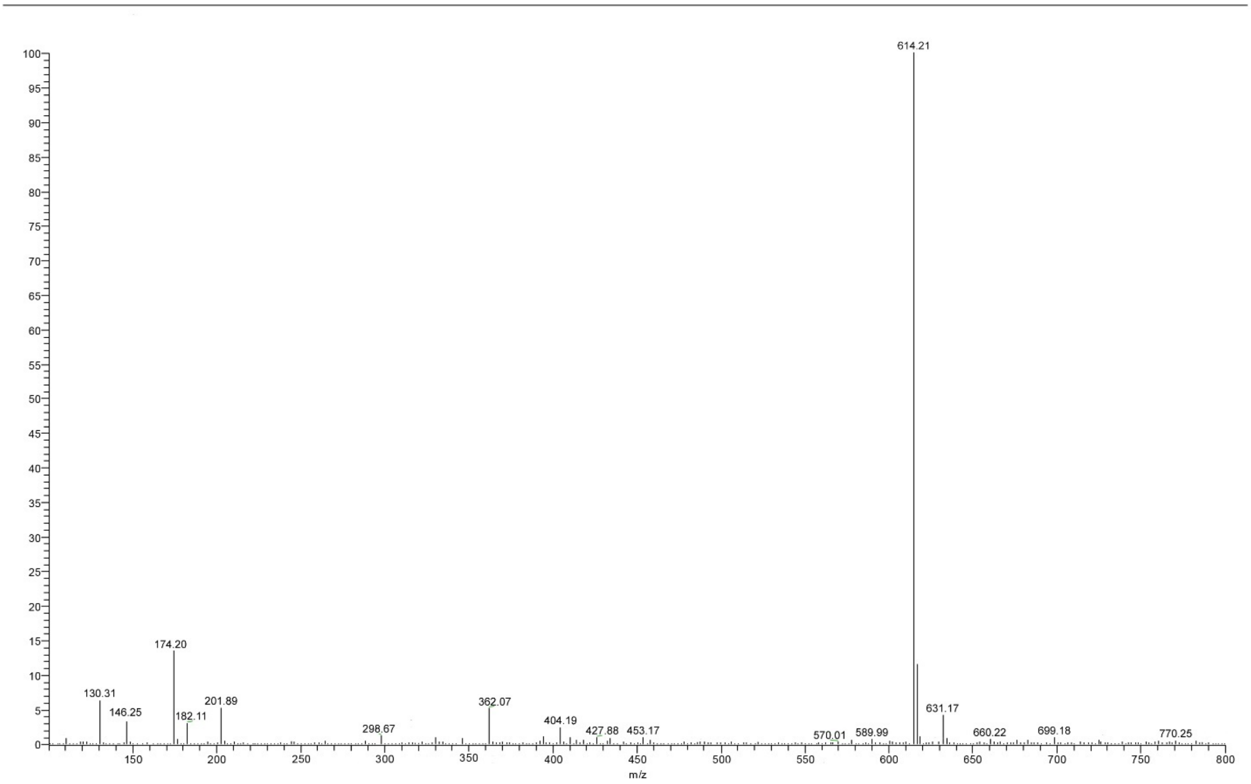

Figure S4. Mass spectrum of 1,1′-bis-dodecyl-2,2′-hexamethylenebispyridinium chloride (GP12_6).

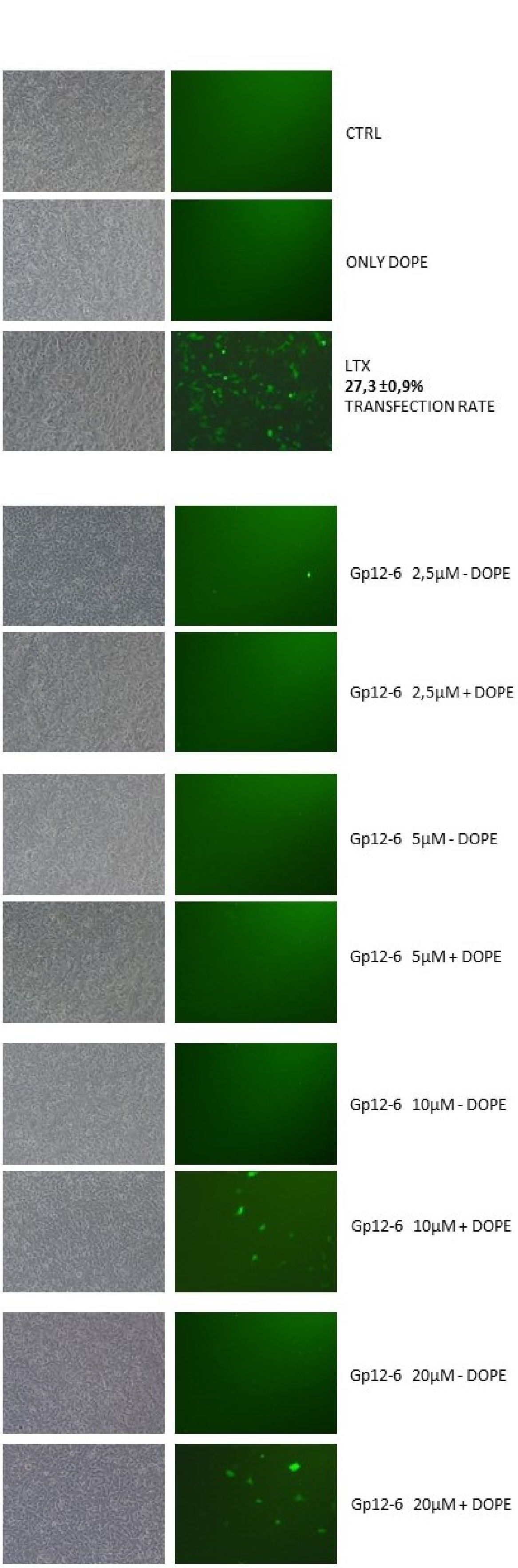

Figure S5. Transfection of RD4 by GP12_6 as a function of concentration. On the left, phase contrast and, on the right, fluorescence microscope observation of the transfected cells (as shown by green cells expressing EGFP) are shown. The experiments were done with the surfactant alone and with surfactant:DOPE=1:2. Cells are not transfected with DOPE alone.

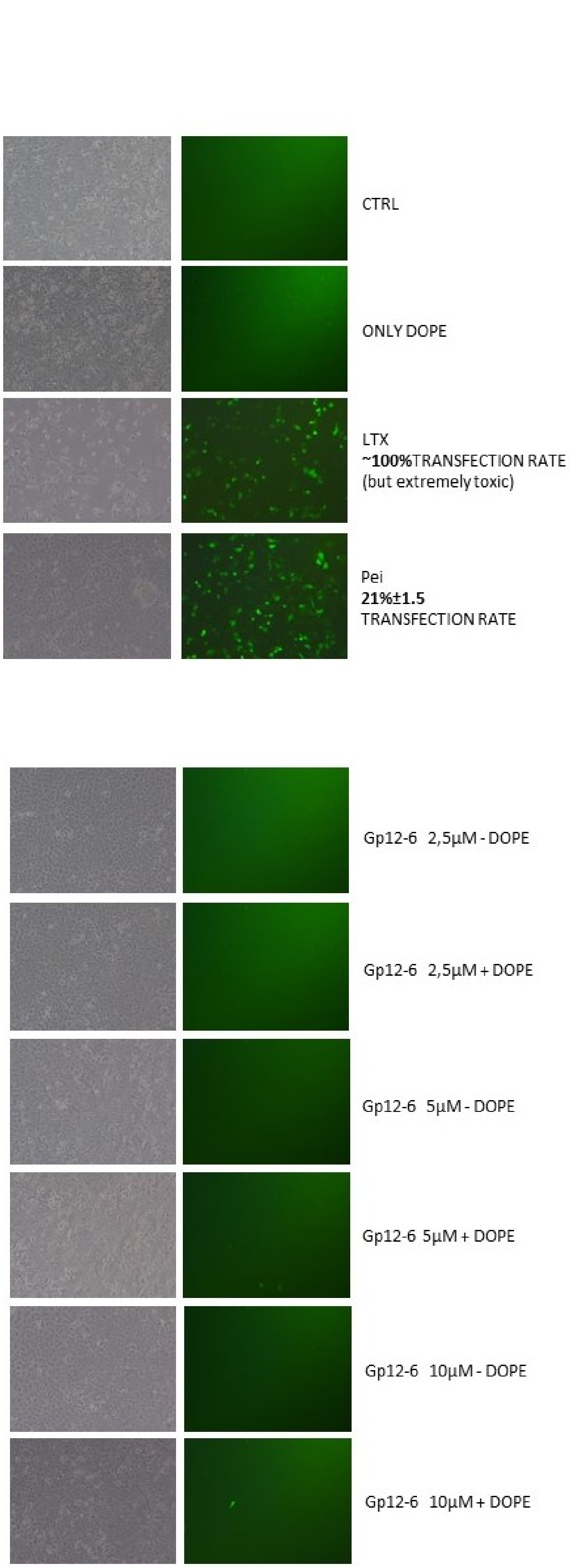

Figure S6. Transfection of A549 cells (right) by GP12_6 as a function of concentration. On the left, phase contrast and, on the right, fluorescence microscope observation of the transfected cells (as shown by green cells expressing EGFP) are shown. The experiments were done with the surfactant alone and with surfactant:DOPE =1:2. Cells are not transfected with DOPE.

DNA

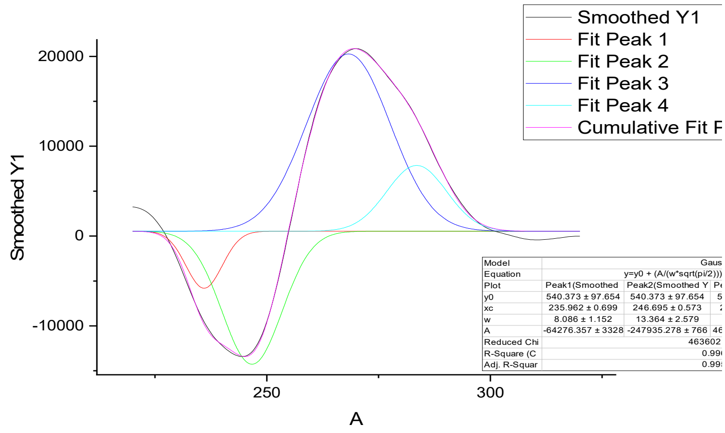

GP12-6 1:4

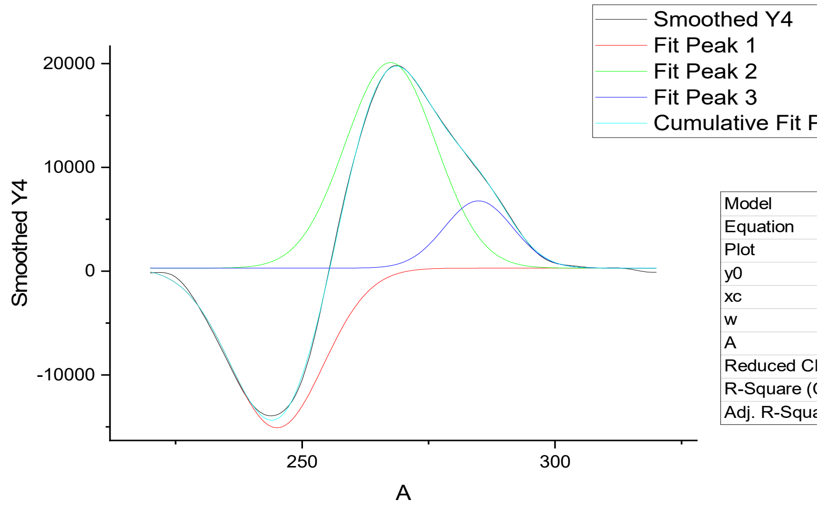

GP16-6 1:2

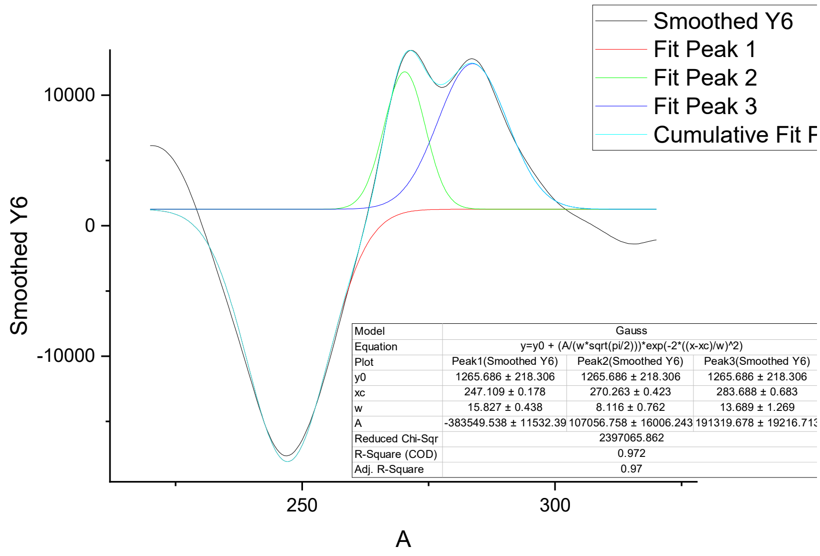

GP16-6 1:4

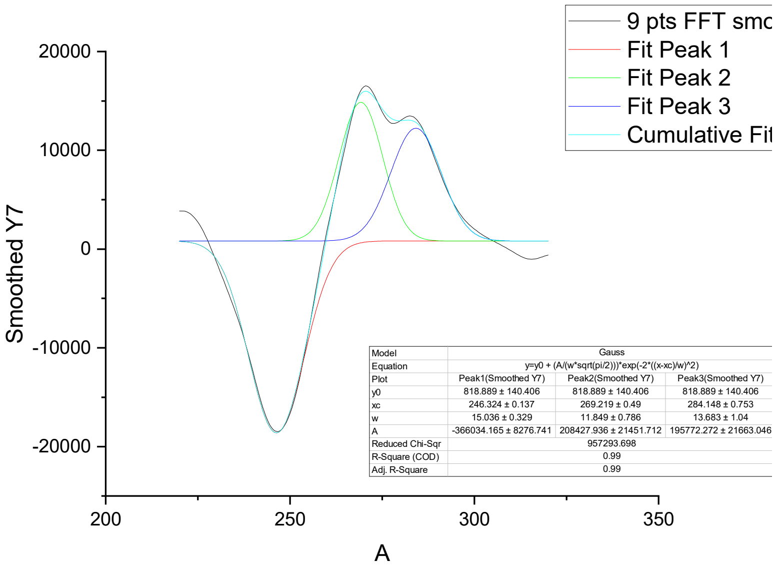

Model	Gauss		
Equation	y=y0 + (A/(w*sqrt(pi/2)))*exp(-2*((x-xc)/w)^2)		
Plot	Peak1(Smoothed Y7)	Peak2(Smoothed Y7)	Peak3(Smoothed Y7)
y0	818.889 ± 140.406	818.889 ± 140.406	818.889 ± 140.406
xc	246.324 ± 0.137	269.219 ± 0.49	284.148 ± 0.753
w	15.036 ± 0.329	11.849 ± 0.786	13.683 ± 1.04
A	-366034.165 ± 8276.741	208427.936 ± 21451.712	195772.272 ± 21663.046
Reduced Chi-Sqr	957293.698		
R-Square (COD)	0.99		
Adj. R-Square	0.99		

FGP6 1:1

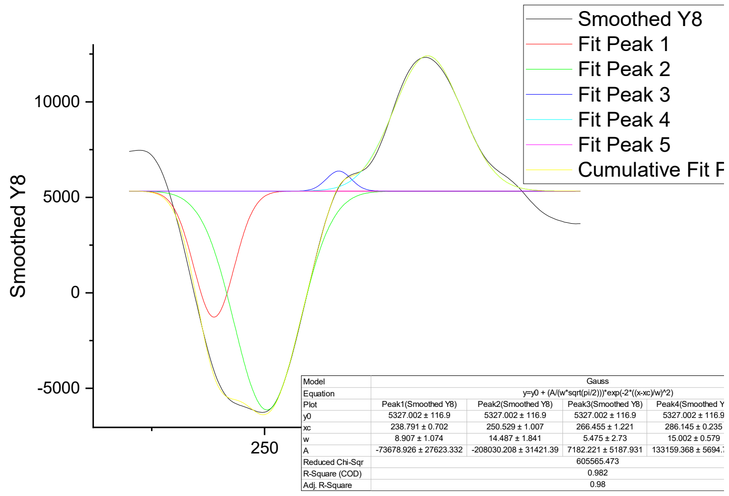

Model	Gauss			
Equation	y=y0 + (A/(w*sqrt(pi/2)))*exp(-2*((x-xc)/w)^2)			
Plot	Peak1(Smoothed Y8)	Peak2(Smoothed Y8)	Peak3(Smoothed Y8)	Peak4(Smoothed Y
y0	5327.002 ± 116.9	5327.002 ± 116.9	5327.002 ± 116.9	5327.002 ± 116.9
xc	238.791 ± 0.702	250.529 ± 1.007	266.455 ± 1.221	286.145 ± 0.235
w	8.907 ± 1.074	14.487 ± 1.841	5.475 ± 2.73	15.002 ± 0.579
A	-73678.926 ± 27623.332	-208030.208 ± 31421.39	7182.221 ± 5187.931	133159.368 ± 5694.[illegible]
Reduced Chi-Sqr	605565.473			
R-Square (COD)	0.982			
Adj. R-Square	0.98			

FGP6 1:2

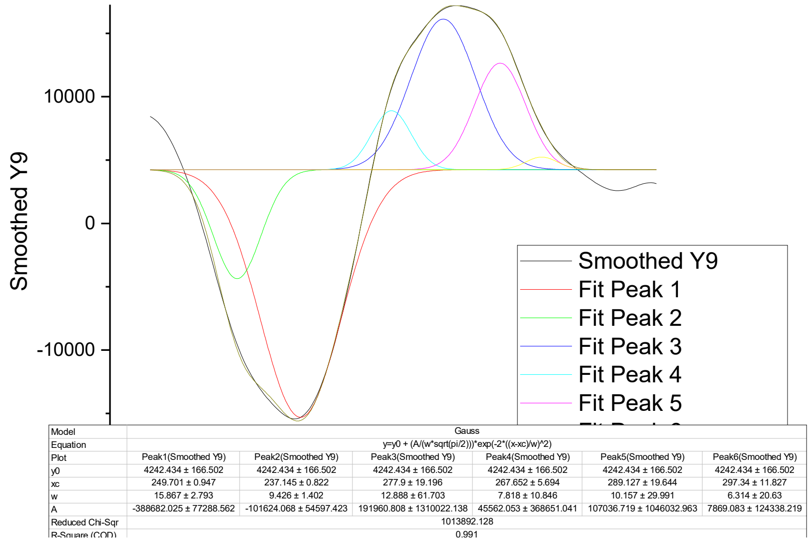

Model	Gauss					
Equation	y=y0 + (A/(w*sqrt(pi/2)))*exp(-2*((x-xc)/w)^2)					
Plot	Peak1(Smoothed Y9)	Peak2(Smoothed Y9)	Peak3(Smoothed Y9)	Peak4(Smoothed Y9)	Peak5(Smoothed Y9)	Peak6(Smoothed Y9)
y0	4242.434 ± 166.502	4242.434 ± 166.502	4242.434 ± 166.502	4242.434 ± 166.502	4242.434 ± 166.502	4242.434 ± 166.502
xc	249.701 ± 0.947	237.145 ± 0.822	277.9 ± 19.196	267.652 ± 5.694	289.127 ± 19.644	297.34 ± 11.827
w	15.867 ± 2.793	9.426 ± 1.402	12.888 ± 61.703	7.818 ± 10.846	10.157 ± 29.991	6.314 ± 20.63
A	-388682.025 ± 77288.562	-101624.068 ± 54597.423	191960.808 ± 1310022.138	45562.053 ± 368651.041	107036.719 ± 1046032.963	7869.083 ± 124338.219
Reduced Chi-Sqr	1013892.128					
R-Square (COD)	0.991					

FGP6 1:4

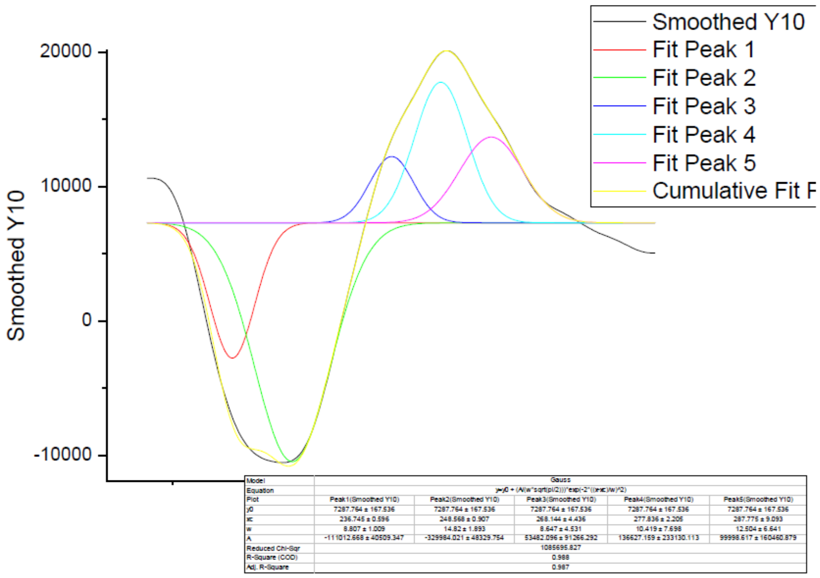

Model	Gauss				
Equation	y=y0 + (A/(w*sqrt(pi/2)))*exp(-2*((x-xc)/w)^2)				
Plot	Peak1(Smoothed Y10)	Peak2(Smoothed Y10)	Peak3(Smoothed Y10)	Peak4(Smoothed Y10)	Peak5(Smoothed Y10)
y0	7287.764 ± 167.536	7287.764 ± 167.536	7287.764 ± 167.536	7287.764 ± 167.536	7287.764 ± 167.536
xc	236.745 ± 0.596	248.568 ± 0.907	268.144 ± 4.436	277.836 ± 2.205	287.775 ± 9.093
w	8.807 ± 1.009	14.82 ± 1.893	8.647 ± 4.531	10.419 ± 7.698	12.504 ± 6.641
A	-111012.668 ± 40509.347	-329984.021 ± 48329.754	53482.096 ± 91266.292	136627.159 ± 233130.113	99998.617 ± 160460.879
Reduced Chi-Sqr	1085695.827				
R-Square (COD)	0.988				
Adj. R-Square	0.987				

Figure S7. Deconvolution of the spectra using Origin Pro, gaussian model.

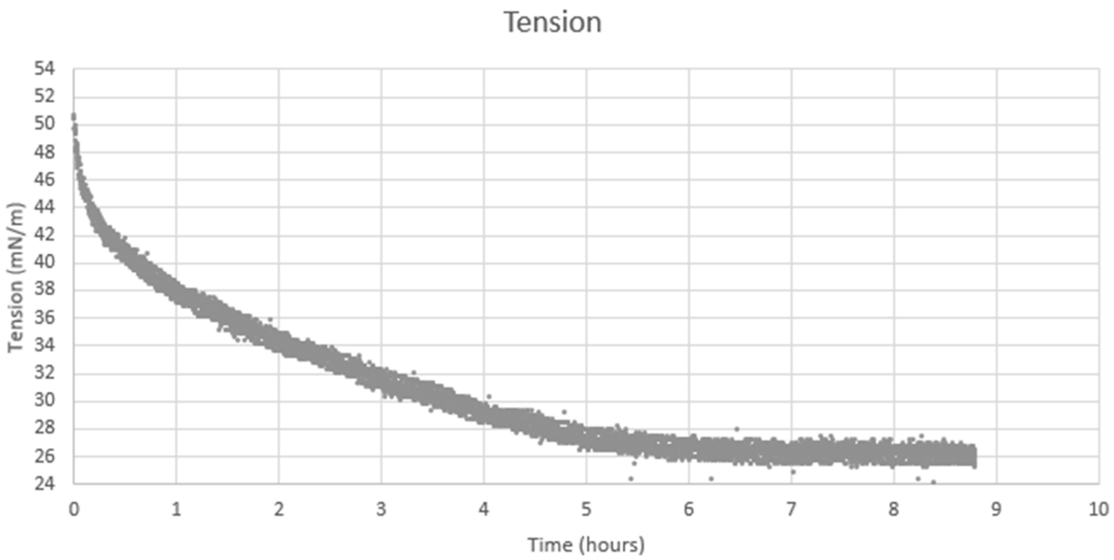

Figure S8. Kinetics of surface layer formation for FGP4 100 µM. It takes about 6 hours to reach the equilibrium. Surface tension is lowered to 26mN/m.

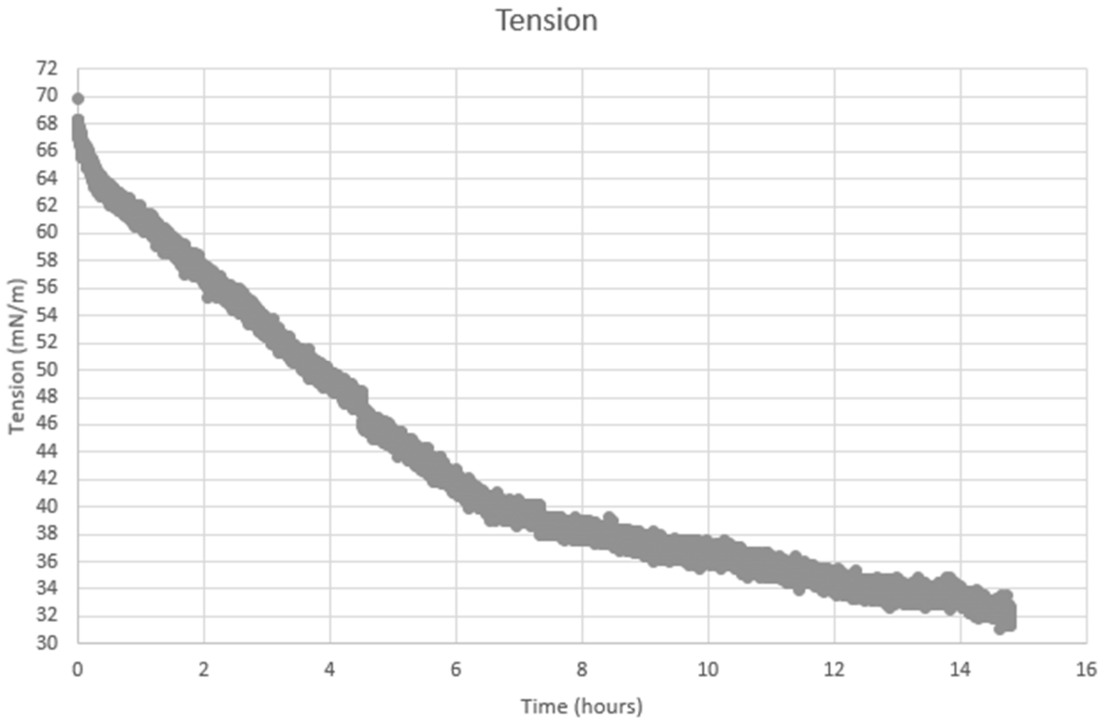

Figure S9. Kinetics of surface layer formation for FGP4 50 µM. After 15 hours, the equilibrium is not reached.

Table S1. Deconvolution of the spectra. DNA: A-form yellow, B-form green.
Wavelength (nm)

**sample**	**A-form**		**B-form**		
	**neg band**	**pos band**	**neg band 2**	**pos band 2**	**pos band 3**
DNA	235.96 ± 0.7	268.25 ± 1.46	246.69 ± 0.57	283.52 ± 2.41	
GP12-6 (1:4)		267.48 ± 0.12	245.03 ± 0.20	284.85 ± 0.39	
GP16-6 (1:2)		270.26 ± 0.42	247.11 ± 0.18	283.69 ± 0.68	
GP16-6 (1:4)		269.22 ± 0.49	246.32 ± 0.14	284.15 ± 0.75	
FGP8-6 (1:1)	238.80 ± 0.71	266.45 ± 1.22	250.53 ± 1.0	286.14 ± 0.23	
FGP8-6 (1:2)	237.15 ± 0.82	267.65 ± 5.69	249.70 ± 0.95	277.9 ± 19.2	289.13 ± 19.64
FGP8-6 (1:4)	236.74 ± 0.6	268.14 ± 4.43	248.57 ± 4.43	277.84 ± 2.2	287.77 ± 9.09
intensity					
**sample**	**A-form**		**B-form**		
	**neg band**	**pos band**	**neg band 2**	**pos band 2**	**pos band 3**
DNA	-5751.1	20219.7	-14035.3	8070.3	
GP12-6 (1:4)		20126.2	-15065.3	6695.6	
GP16-6 (1:2)		14855.7	-18505.9	12296.3	
GP16-6 (1:4)		10302.0	-17605.8	12448.0	
FGP8-6 (1:1)	-1219.4	6373.5	-6120.1	12340.5	
FGP8-6 (1:2)	-4256.0	9009.1	-15231.0	16058.1	12657.6
FGP8-6 (1:4)	-2810.6	12191.8	-10363.0	17760.7	13681.8

Table S2. Surface tension values of FGP4, FGP6 and FGP8 after 2 hours. . For each concentration, the mean and its relative positive and negative error are reported.

**Concentrations**	**FGP4**			**FGP6**			**FGP8**		
	**Mean**	**Error +**	**Error -**	**Mean**	**Error +**	**Error -**	**Mean**	**Error +**	**Error -**
**2.5**	68.32	69.1	67.54	64.1	64.67	63.53	63.52	64.27	62.77
**5**	69.1	69.77	68.43	61.135	61.86	60.41	62.895	63.31	62.48
**10**	62.7	63.2	62.2	56.44	57.07	55.81	55.04	55.78	54.3
**20**	61.515	62.06	60.97	52.62	53.46	51.78	46.27	47.02	45.52
